# A Survey on Learning Objects' Relationship for Image Captioning

**DOI:** 10.1155/2023/8600853

**Published:** 2023-05-29

**Authors:** Du Runyan, Zhang Wenkai, Guo Zhi, Sun Xian

**Affiliations:** ^1^Aerospace Information Research Institute, Chinese Academy Sciences, Beijing, China; ^2^University of Chinese Academy of Sciences, Beijing, China; ^3^School of Electronic, Electrical and Communication Engineering, University of Chinese Academy of Sciences, Beijing, China; ^4^Key Laboratory of Network Information System Technology, Aerospace Information Research Institute, Chinese Academy of Sciences, Beijing, China

## Abstract

Image captioning is a challenging modality transformation task in computer vision and natural language processing, aiming to understand the image content and describe it with a natural language. Recently, the relationship information between objects in the image has been investigated to be of importance in generating a more vivid and readable sentence. Many types of research have been done in relationship mining and learning for leveraging into the caption models. This paper mainly summarizes the methods of relational representation and relational encoding in image captioning. Besides, we discuss the advantages and disadvantages of these methods and provide commonly used datasets for the relational captioning task. Finally, the current problems and challenges in this task are highlighted.

## 1. Introduction

Image captioning[1–30] is to understand the content of an image and further inference a natural sentence to describe it. The generated description needs to achieve satisfactory accuracy, adequacy, and readability [9, 31–33]. Readability requires the sentences to satisfy grammatical rules, the accuracy makes the content of generated sentences conform to the content of images, and the adequacy measures the adequacy of the generated sentences to express the image information. The adequacy and accuracy of the sentence include whether the visual vocabulary (describing the category and attributes of the object) and the relational vocabulary (describing the relationship between the objects) are fully reflected and whether they conform to the image's content.

The early captioning methods theoretically use image-to-text retrieval [1, 34] or filling sentence templates [35–37] to improve the adequacy and accuracy of the generated sentences. In technical, they mainly use the static object categories and the statistical language model. In technical, they mainly use the static object categories and the statistical language model. About retrieval methods, Aker and Gaizauskas [34] used a dependency model to summarize the information contained in multiple web documents and localize this information to images. Kulkarni et al. [1] used conditional random fields based on the objects detected in the image to predict the image's label for retrieval. About templates' methods, Li et al. [35] proposed a network-scale-basedn-gram method to collect candidate phrases and other form sentences. Yang et al. [36] proposed a language model trained on the English Gigaword corpus to obtain the action in the image and incorporated them into a hidden Markov model. Lin et al. [37] used a 3D visual analysis system to represent objects, attributes, and relationships in images. They transformed them into a series of semantic trees, from which they learned grammar and generated sentences.

However, the early captioning methods [1, 34–37] are suffered from few shortcomings. The template-based methods would make the generated sentences rigid and lack readability. At the same time, the retrieval would lead to mismatches between images and texts, affecting accuracy or adequacy. With the development of the deep learning technology [38–49], Vinyals et al. [2] proposed an encoder-decoder model, which uses convolutional neural networks [40] to understand objects and scenes in images, and uses LSTM [44, 50] to model the long-term dependency between words. Specifically, the generation of individual words in a sentence depends on the memory state and the image's global information. Xu et al. [3] incorporated an attention mechanism with the encoder-decoder framework to align text to specific regions in an image. Lu et al. [4] proposed an adaptive attention method that utilizes visual sentinels to align nonvisual vocabulary during sentence generation. In the related multimodal field [51–57], Ding et al. [58] introduced the attention mechanism to the video captioning, so that the model can adaptively focus on the elements, parts, or details in the image when dealing with each frame. Qin et al. [59] considered the visual coherence of the attention region and introduced the memory ability in the attention mechanism. For alleviating the accumulated error on sentence generation, they proposed a new language model which generates sentence chunks by chunks instead of words-by-words.

Furthermore, to more accurately align objects with words, Anderson et al. [5] adopted an object detection network to detect objects and constructed a two-LSTMs' decoder to learn the dependencies between words in sentences and the alignment between words and image regions. For enhancing the vocabulary coherence between words and syntactic paradigm of sentences, Ke et al. [60] proposed a new LSTM variant which considered the previous generated words and their relative positional information during decoding. This perception can also bring great improvement when integrating it with the image captioning models. Ding et al. [61] were inspired by the perception of the human brain and adjust the attention weight of each object according to its own color, area of bound box, and visual permutations.

In recent years, with the development of full-attentive models [9, 14, 18, 62, 63], Vaswani et al. [64] proposed the Transformer to use attention to learn interactions of intermodality and intramodality. They obtained excellent achievements in natural language processing, such as machine translation. Zhu et al. [6] applied transformer to image captioning and confirmed the effectiveness of the transformer in the captioning task. The transformer learns the interrelationships between object attribute features in visual sequences through the encoder and utilizes attention in the decoder to align text features with visual features. Under the object features [38, 65] provided by the pretrained object detection network [38, 43, 65], the accuracy and adequacy of the visual vocabulary generation are significantly improved with the reinforcement learning strategy [12, 66]. On the other hand, BERT-based vision-language pretraining methods [67, 68] concentrate on designing a unified framework for multiple vision-language tasks, which first optimize the object's features by specific pretraining objectives and then generating sentence after finetuning the features with the caption objective. Those methods have achieved a new higher-level performance in image captioning. Furthermore, Li et al. [69] have designed a decoupled encoder-decoder framework with a scheduled sampling strategy for countering the incompatibility between VL understanding and caption generation. Recently, Li et al. [70] have used the cross-modal retrieval technique to generate a primary sentence and refine its content with the transformer blocks, which extremely improved the model performance in the end-to-end training mode. In order to have a better caption development, a unified codebase [71] has been proposed which covered many high performance modules in each stage of the cross-modal analytics between vision and language in the multimedia field.

**Figure 1:**
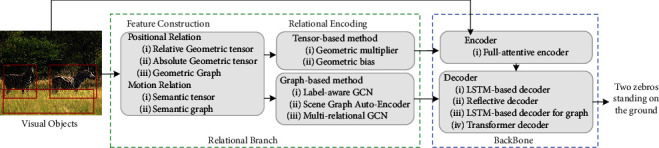
The taxonomy of the visual relationship.

Since 2019, some studies [62, 63, 72–74] have begun to focus on characterizing the relationship between objects based on the abovementioned works to improve the generation of relational vocabulary. For modeling the objects' relationships, researchers first start from the basic spatial relationship to explicitly perceive relational information and establish alignment with relational words. Then, they take a far more step to mine the higher-level semantic relationships hiding in the image. In this process, low-level geometric spatial features are less difficult to be constructed, but the constructed features are also less capable of representing complex relationship categories in textual modality. The relationship between objects can be reflected by multiple relationship categories with similar meanings, which belong to multirelational data. In the case of multirelational data in images, finding higher-level relational features is a difficult challenge. After feature construction, how to effectively combine relational features in the feature optimization stage so that the optimized features can have good separability for different relational categories is a problem worth studying. In order to follow up the development of relational image captioning, it is necessary to overview the previous works about relationships and assist the following researchers in improving the intelligence of captioning models. This paper mainly classifies and summarizes the extraction methods of this relational information and their corresponding encoding methods in the current image captioning. According to the frame shown in Figure 1, we overview the main line of relational captioning and summarize a taxonomy of relational methods. Meanwhile, the commonly used datasets and evaluation measures are available in this paper. The advantages and disadvantages of methods and future development prospects are analyzed.

### 1.1. Contributions

Our contributions in this paper are shown as follows:

1. Combining all previous studies in relational image captioning, we summarize a taxonomy of relational information processing in the image, which includes feature construction and encoding. Meanwhile, we introduce the corresponding methods and analyze their strength and weakness.
2. We review the relevant datasets involved in the relational image captioning, covering relational understanding and image captioning datasets. The metrics used in evaluation are also recorded in this paper.
3. We observe and analyze the development of the relational image caption and enumerate the main challenges in this area and future development directions.

This paper is organized as follows: the second section briefly introduces the content of the visual branch in relational captioning, mainly about the basic knowledge and overall framework commonly used in the relational image description. The third section explicitly describes the construction of relational features in images. The fourth section mainly describes the encoding of relational information. The fifth section mainly describes the datasets and related evaluation indicators used to extract and learn relational data in image captioning. The sixth section concludes and presents the prospect of future development in this field.

## 2. Backbone

The backbone of relational captioning is the standard encoder-decoder framework [2–4] as the common captioning task. It is irrelevant to the relationship but is necessary to discuss for constructing the whole procedure. As shown in Figure 1, the backbone consists of two parts: encoder and decoder. Given an image *I*, relational captioning begins with objects detected from the object detector [38]. The encoder refines each element in the visual sequence and further feed it into the decoder for generating a natural sentence.

### 2.1. Encoder

#### 2.1.1. Full-Attentive Encoder

**Figure 2:**
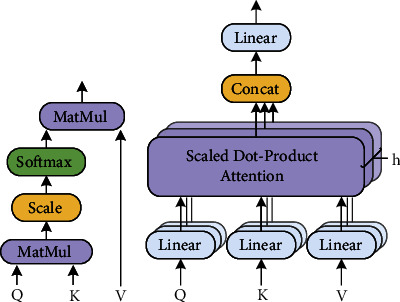
The scaled dot-product attention and multihead attention.

Initializing from the visual sequence *𝒱*={*v*_1_, *v*_2_,…, *v*_*n*_}, the purpose of the encoder is to enrich each object's feature. Recently, transformer-dominated full-attentive models [2] play an important role in relational captioning. The most important component in transformer is the scaled dot-product attention operator, whose structure is shown in Figure 2(a). Its calculation formula is shown as follows:

(1) $$\begin{array}{c} Att\left(Q,K,V\right)=softmax\left(\frac{QK^{T}}{\sqrt{d}}\right)V\text{.} \end{array}$$

It calculates the similarity of each query vector *q* ∈ *R*^*d*^ in the query matrix **Q** ∈ *R*^*N*×*d*^ and each key vector in the key matrix **k** ∈ *R*^*d*^. The generated attention weight *E*=**QK**^**T**^. *E* is multiplied with **V** so that each output vector comes from a weighted sum of each element in **V** and its corresponding weight in the weight matrix. Meanwhile, to further enhance the model representation ability of the attention operator [64] and speed up the convergence of the model during the training process, the multihead attention mechanism [64] is combined with the conventional attention operator, as shown in (b) in Figure 2. Its formula is calculated as follows:

(2) $$\begin{array}{c} MAtt\left(Q,K,V\right)=\underset{i=1:h}{Concat}\left(Att\left(Q_{i},K_{i},V_{i}\right)\right)\text{.} \end{array}$$

*i* is the index of each head. Each head is a segmentation of the original feature space. The dimension of each subspace is *d*/*h*, where *h* is the number of total heads. The multihead attention mechanism performs self-attention calculations in each subspace and further fuse all outputs from each subspace with Concat. After passing through the encoder, the optimized sequence of object features is fed into a subsequent decoder to generate sentences.

### 2.2. Decoder

#### 2.2.1. LSTM-Based Decoder

Decoders for relational captioning are various language models, commonly using LSTM [44], transformer, and their variants. We denote the output of the encoder as *𝒳*={*x*_1_, *x*_2_,…, *x*_*n*_}. Given *𝒳*, Anderson et al. [5] build a decoder with two LSTMs, which contain an attention LSTM and a language LSTM, respectively. The attention LSTM takes the word embedding vector *w*_*t*−1_ and the hidden layer state of the language LSTM *h*_*t*−1_^*l*^ at the last moment and the global visual feature (average of all object features) $\bar{g}$ as the input to calculate the current moment's hidden layer state *h*_*t*_^*a*^.

(3) $$\begin{array}{c} h_{t}^{a}=LSTM\left(\left[\bar{g};w_{t-1};h_{t-1}^{l}\right],h_{t-1}^{a};\theta^{a}\right)\text{,}\\ {\tilde{\alpha }}_{t,i}^{c}=w_{c}^{T}\tanh\left(W_{xc}x_{t,i}+W_{hc}h_{t}^{a}\right)\text{,}\\ \alpha_{t}^{c}=softmax\left({\tilde{\alpha }}_{t}^{c}\right)\text{.} \end{array}$$

As an attention query, *h*_*t*_^*a*^ computes the attention score ${\tilde{\alpha }}_{t,i}^{c}$ with each element of *𝒳*. The *α*_**t**_^**c**^ is the context attention weight for fusing *𝒳* into a context vector. The language LSTM takes the current hidden state *h*_*t*_^*a*^ of attention LSTM and the context vector to generate the current word representation *w*_*t*_.

#### 2.2.2. Reflective Decoder

In the word-by-word decoding process, modeling the previous content and the positional information of each word is beneficial for generating words in the current time step. Ke et al. [60] enhance the LSTM-based decoder with reflective attention and reflective position modules. In the LSTM-based decoder, the output of language LSTM *h*_*t*_^*l*^ is followed by a linear function for generating the current word. In the reflective attention module, it replaces *h*_*t*_^*l*^ with an attended result ${\hat{h}}_{t}^{l}$ reasoned by the previous generated content.

(4) $$\begin{array}{c} \alpha_{i,t}^{ref}=W_{h}^{l}\tanh\;\left(W_{h_{2}}^{l}h_{i}^{l}+W_{h_{1}}^{l}h_{t}^{a}\right)\text{,}\\ \alpha_{t}^{ref}=softmax\left(a_{t}^{ref}\right),a_{t}^{ref}={\alpha_{i,t}^{ref}}_{i=1}^{t}\text{,} \end{array}$$

where *α*_*i*,*t*_^ref^ is the attention weight corresponding to each *h*_*i*_^*l*^ in *i*-th time step. Besides, ${\hat{h}}_{t}^{l}$ is constrained by the relative position of each word in the sentence with a loss function which minimizes the distance between ${\hat{h}}_{t}^{l}$ and *t*/*n*, where *t* is the time step of each word and *n* is the length of the sentence.

#### 2.2.3. LSTM-Based Decoder for Graph

For introducing the graph structure into the language decoder, Chen et al. [74] proposed a variant of a conventional two-LSTMs decoder which consists of two modules: graph-based attention mechanism and graph update mechanism. The graph-based attention mechanism computes two attention weights: *α*_**t**_^**c**^ and *α*_**t**_^**f**^. *α*_**t**_^**c**^ is the context attention weight which follows the two-LSTMs decoder. *α*_**t**_^**f**^ is the flow attention weight which constrains the model to attend the semantically relevant node within the neighbors of the previous attended one. Specifically, it is a soft interpolation of the three flow scores with a dynamic gate. According to the different moving steps, the three flow scores are computed with the adjacency matrix **M**_**f**_: (1) stay at the same node *α*_**t**,0_^**f**^=*α*_**t**−1_, (2) move one step *α*_**t**,0_^**f**^=**M**_**f**_*α*_**t**−1_, and (3) move two steps *α*_**t**,2_^**f**^=(**M**_**f**_)^2^*α*_**t**−1_. The flow attention is computed as follows:

(5) $$\begin{array}{c} s_{t}=softmax\left(W_{s}\sigma \left(W_{sh}h_{t}^{a}+W_{sz}z_{t-1}\right)\right)\text{,}\\ \alpha_{t}^{f}=\overset{2}{\underset{k=0}{\sum }}\alpha_{t,k}^{f}\text{,}\\ \beta_{t}=sigmoid\left(w_{g}\sigma \left(W_{gh}h_{t}^{a}+W_{gz}z_{t-1}\right)\right)\text{,}\\ \alpha_{t}=\beta_{t}\alpha_{t}^{c}+\left(1-\beta_{t}\right)\alpha_{t}^{f}\text{.} \end{array}$$

The final attention weight *α*_**t**_ takes a balance between *α*_**t**_^**c**^ and *α*_**t**_^**f**^ with a gate function. To avoid repetition and omission in the attention process, Chen el al. [74] use a graph update mechanism to dynamically remove or preserve some nodes with a visual sentinel **u**_**t**_.

(6) $$\begin{array}{c} u_{t}=sigmoid\left(f_{vs}\left(h_{t}^{l};\theta_{vs}\right)\right)\alpha_{t}\text{.} \end{array}$$

The scalar **u**_**t**,**i**_ indicates whether the generated word expresses the attended node. For avoiding repetition, an erase gate for the *i*-th node *e*_*t*,*i*_ is computed according to its visual sentinel *u*_*t*,*i*_. Meanwhile, if a node needs multiple access, an add gate for the *i*-th node *a*_*t*,*i*_ is also computed to preserve its status.

(7) $$\begin{array}{c} e_{t,i}=sigmoid\left(f_{ers}\left(\left[h_{t}^{l};x_{t,i}\right];\theta_{ers}\right)\right)\text{,}\\ {\hat{x}}_{t+1,i}=x_{t,i}\left(1-u_{t,i}e_{t,i}\right)\text{,}\\ a_{t,i}=\sigma \left(f_{add}\left(h_{t}^{l};x_{t,i};\theta_{add}\right)\right)\text{,}\\ x_{t+1,i}={\hat{x}}_{t+1,i}+u_{t,i}a_{t,i}\text{,} \end{array}$$

where *f*_*∗*_ are fully connected networks and *θ*_*∗*_, *W*_*∗*_, and *w*_*∗*_ are the learnable parameters.

#### 2.2.4. Transformer Decoder

The transformer decoder proposed by Vaswani et al. [64] is also widely used in image captioning, which consists of multiple sublayers. The textual features in each sublayer first learn the interaction within its modality through self-attention, then align specific object features through the cross attention between the textual features and *𝒳*. They finally pass the fully connected layer to generate the representation *w*_*t*_ of the word at the current moment. *w*_*t*_ finally generates the corresponding word through the mapping matrix and the softmax function.

In summary, relational image description's overall process is generating sentences through the visual branch. At the same time, the relational branch processes the object-level relational features to be integrated into the visual branch. In the vision branch, given an image *I*, the object feature sequence *𝒱* obtained by target detection is used as input, and then *𝒳* is obtained by encoder learning. The commonly used models in encoders are mainly transformer encoders or graph convolutional networks [72–76]. Then, *V*_*e*_ is input to the transformer decoder or double LSTM to generate natural sentences word-by-word.

## 3. Relational Branch

**Figure 3:**
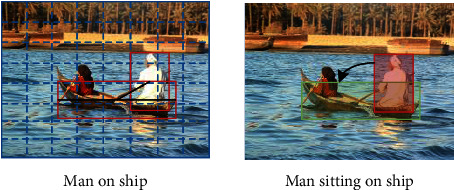
The example for illustrating the positional relation and motion relation.

The relational branch is the core of relational captioning. It concentrates on the encoder part and incorporates the relationship between objects into the encoder. It includes two steps:(1) feature construction and (2) relational encoding. The relationships in image can be divided into two categories: (1) position relationships and (2) action relationships, corresponding to the positional words and predicate words. As shown in Figure 3, the position relationship refers to the geometric relationship between the objects, which can be expressed as positional words in sentences, such as “in” and“ on.” On the other side, the action relationship represents more complicated and higher-level semantic relationship between the subject and the object. In textual modality, a predicate generally represents one kind of action relationship, As shown in Figure 3. This section mainly introduces different relational feature construction methods and feature encoding methods according to the different types of relations.

### 3.1. Feature Construction

The first step in relational captioning is extracting and constructing relational features. Many studies have explored the relationship between objects in images in visual relationship detection and scene understanding. The position relationship represents the up-down, left-right relationship between two objects in the 2-dimensional space. It corresponds to the words describing the position in the sentence, such as “on” and “near”. The action relationship between objects represents a specific action, which is corresponding to a particular predicate verb in the generated sentence. Figure 3 defines the abovementioned two kinds of relationships. In this section, we mainly summarize the current extraction methods of these two kinds of relational information and list the advantages and disadvantages of each technique.

#### 3.1.1. Positional Relationship

The positional relationship between objects is usually represented by the geometric relationship between two objects' bounding boxes in two-dimensional space. Given an image *I* and *N* object boxes in it, the position vector of each object box is represented as (*x*_*i*_, *y*_*i*_, *w*_*i*_, *h*_*i*_), and the geometric relationship between the object boxes includes the relative distance, relative angle, and relative area between the object boxes. According to the different data structures, the representation methods can be divided into two types: (1) tensor and (2) graph.

#### 3.1.2. Relative Geometric Tensor

The main idea is to construct a *N* × *N* × *d* tensor to represent all *N* × *N* object pairs. Each of these relations is a *d*-dimensional vector. Herdade et al. [62] and Guo et al. [63] used the relative distances of the box's center and relative size ratios between objects' boxes to construct geometric vectors:

(8) $$\begin{array}{c} \left(\log\left(\frac{\left|x_{j}-x_{i}\right|}{w_{i}}\right),\log\left(\frac{\left|y_{j}-y_{i}\right|}{h_{i}}\right),\log\left(\frac{w_{j}}{w_{i}}\right),\log\left(\frac{h_{j}}{h_{i}}\right)\right)\text{.} \end{array}$$

The subscripts *i* and *j* represent the image's *i*-th and *j*-th objects. The external logarithmic function plays a numerically stable role in ensuring that when the width and height of the object box *i* are very small. The output value will not be too far away from the mean value, resulting in excessive variance and making the model difficult to converge. All the *N* × *N* object pairs' geometric vectors form the *N* × *N* × 4 geometric tensor. Meanwhile, the activation ReLU filters the negative elements when two objects' boxes are very close.

In summary, the geometric feature mainly describes the relative distance between the center points of the two object boxes and the relative size ratio between the object boxes. It can provide basic prior information about the object's size and location, which is very helpful for image understanding. However, the geometric features extracted by this method are not enough to represent high-level semantic relationship categories, and they are also interfered by the scale information of the bounding box when representing different spatial orientations, that is, the amount of relationship that needs to be calculated is large, and all object pairs in the image need to be considered in the calculation process. In practical use, if a complex network model is constructed to learn geometric feature tensors, it often brings a lot of computational costs. To a certain extent, the learning ability of the model for the position relationship information between objects is limited.

#### 3.1.3. Absolute Geometric Tensor

The absolute geometric tensor directly maps the coordinates of the object frame in the image to the feature space. Luo et al. [77] designed a transformer variant for processing grid features and object features and used an absolute geometric tensor to encode the positional information of each grid in the feature map. It is represented by the concatenation of two 1-*d* sine and cosine embeddings:

(9) $$\begin{array}{c} GPE\left(i,j\right)=\left[PE_{i};PE_{j}\right]\text{,}\\ PE\left(pos,2k\right)=\sin\left(\frac{pos}{10000^{2k/\left(d/2\right)}}\right)\text{,}\\ PE\left(pos,2k+1\right)=\cos\left(\frac{pos}{10000^{2k/\left(d_{model}/2\right)}}\right)\text{,} \end{array}$$

where *i* and *j* are the row and column indices of the grid, respectively, and *PE*_*∗*_ is the position encoding vector of the *d*/2 dimension. pos is the corresponding position, and *k* is each dimension. For object features, it directly maps the coordinates to the feature space. Its formula is as follows:

(10) $$\begin{array}{c} RPE\left(i\right)=B_{i}W_{emb}\text{,} \end{array}$$

where *B*_*i*_ = (*x*_min_, *y*_min_, *x*_max_, *y*_max_) are the coordinates of the upper left corner and lower right corner of the object bounding box. *W*_emb_ is the embedding matrix. Absolute geometric features are geometric features aimed at fixed image regions, which can effectively improve the spatial separability of features, but they lack flexibility.

#### 3.1.4. Geometric Graph

The data structure of a graph can naturally use edges to represent the relationship between nodes. Therefore, using the graph to represent the relationship in relational captioning is natural. Specifically, for the graph structure data *G*=(*V*, *E*), its composition includes the node set *V* and the edge set *E*. Each node corresponds to an object in the image. In related tasks in the multimodal field, nodes generally contain corresponding node features, and the representation matrix of all nodes in the node set is *X* ∈ *R*^*n*×*d*^. In addition to the nodes, each edge in the edge set is represented as *e*_*ij*_=(*v*_*i*_, *v*_*j*_) ∈ *E*. At the same time, if edge features are required, all edge feature matrices are *X*^*e*^ ∈ *R*^*m*×*c*^, where the feature of each edge between *i*-th and *j*-th objects is a *c*-dimensional vector *X*_*i*,*j*_^*e*^ ∈ *R*^*c*^.

Since the edge represents the relationship between two objects, it can be expressed formally as follows: <subject-relation-object>, where subject indicates that the subject-object corresponds to *v*_*i*_, an object indicates that the object corresponds to *v*_*j*_. The neighbors of a node *v* can be expressed as *N*(*v*)={*u* ∈ *V*|(*v*, *u*) ∈ *E*}. Its adjacency matrix *A* is a matrix of *n* × *n*, where *A*_*ij*_=1 if *e*_*ij*_ ∈ *E*, *A*_*ij*_=0 if *e*_*ij*_ ∉ *E*.

**Figure 4:**
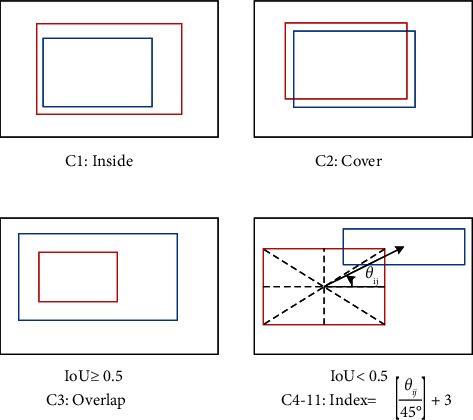
The discretization of positional relation of each object's pair. The bounding boxes of subject and object are marked with 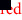 and 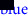, respectively.

**Figure 5:**
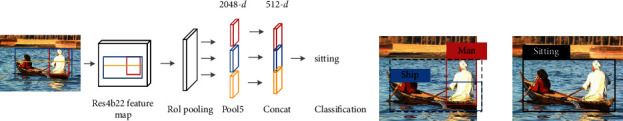
(a) The left part infers the corresponding relationship labels from the pretrained relationship detection network and (b) the right part represents the specific relationship through the feature of the union box between the two objects.

One approach to embedding relational information into the edges is to classify the positional relation and assign it as a label to each edge. Yao et al. [72] discretized the positional relationship based on the geometric features between two objects' boxes and assigned categories to each edge to build a directed graph. Specifically, according to the difference in the positional relationship between the two object boxes, they can be divided into 11 categories, as shown in Figure 4. Specifically, categories 1 and 2 are the inclusion and included relationships between the subject and the object, respectively. Category 3 is the overlapping relationship between the two objects with their IoU greater than or equal to 0.5. The remaining categories are divided into 8 categories according to the relative angle between the center points, representing 8 different positions, respectively. After classifying the positional relationship into a number of specific categories, the corresponding label is further assigned to each edge to construct the graph. An example of its graph structure is shown in Figure 5(a), which belongs to a directed fully connected graph. The feature corresponding to each edge is a specific category of positional relationship.

In summary, the graph-based approach can naturally utilize the adjacency matrix to characterize the relationship between objects. The graph is more interpretable and controllable than the tensor method. The tensor method is equivalent to processing an undirected fully connected graph when it uses full attention for subsequent learning. However, the relational content represented by each edge in the graph still depends on a small number of spatial categories, which result in poor performance in representing complex relational words in sentences.

### 3.2. Motion Relationship

The action relationship between objects is more specific than the positional relationship, which reflects the relationship at a higher semantic level. With the different data structures, the motion relation can also be divided into the following two forms: (1) tensor and (2) graph. The first method is more intuitive. The complexity of the motion relation makes it difficult to represent by the geometric feature. Therefore, many studies [73, 74, 78–81] begin to directly mine the information from the image content, extract the features of relevant image regions, and represent them in the form of tensor. The second method uses the graph pretrained by the upstream tasks to generate a suitable graph.

#### 3.2.1. Semantic Tensor

Given an image and its *N* objects, the motion relation is represented in the form of a *N* × *N* × *d* tensor. Specifically, for the action relationship between object *i* and object *j*, the tensor-based method attempts to extract the union content of the two objects in the image to represent the corresponding relationship. The extracted image area must contain two objects' bounding boxes simultaneously to ensure that the extracted content contains an accurate action relationship and avoid other noises as much as possible. The image region from which Zhang et al. [82] extracted features is the minimum circumscribing moment of the two object boxes, as shown in Figure 5. Specifically, for the coordinate (*x*_*i*_, *y*_*i*_, *w*_*i*_, *h*_*i*_) of the object *i* and the space coordinate vector (*x*_*j*_, *y*_*j*_, *w*_*j*_, *h*_*j*_) of the object *j*, the coordinate of the union box is follows:

(11) $$\begin{array}{c} \min\;\left(x_{i}-\frac{w_{i}}{2},x_{j}-\frac{w_{j}}{2}\right),\min\left(y_{i}-\frac{h_{i}}{2},y_{j}-\frac{h_{j}}{2}\right)\text{,}\\ \max\;\left(x_{i}+\frac{w_{i}}{2},x_{j}+\frac{w_{j}}{2}\right),\max\;\left(y_{i}+\frac{h_{i}}{2},y_{j}+\frac{h_{j}}{2}\right)\text{.} \end{array}$$

The union image area passes through the pretrained convolutional network to obtain the corresponding features. Each image can obtain a relation matrix of *N* × *N* × *d* for different downstream tasks.

In summary, the tensor-based method stores the image features that characterize each relational region into relational tensors for the subsequent learning of relational information. This method is relatively straightforward, but it inevitably introduces noise. The noise here refers to relational information that is irrelevant to the relation contained in the generated sentence. At the same time, in general, there are many objects obtained by object detection. In the image description task, the model directly calculates all *N* × *N* relational features will bring a lot of computational costs. In terms of model performance, the quality of generated sentences is determined by the extracted features, which further depend on the structure of the pretrained convolutional network and its training objectives in upstream tasks. This leads to researchers needing to spend more energy on additional tasks. At the same time, after considering the additional pretrained network, the caption model is more computationally intensive overall.

#### 3.2.2. Semantic Graph

**Figure 6:**
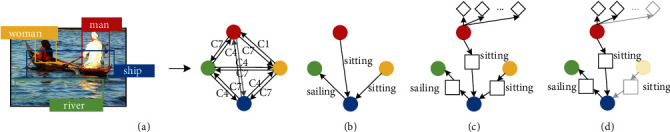
Different types of graph structures are used when modeling the relationship between objects in an image. From the left to right, respectively, (a), (b), (c), and (d).

The graph method use pretrained relationship detection networks in visual relation detection to extract action relations between objects and construct corresponding scene graphs. Specifically, Yao et al. [72] used the abovementioned method to build the graph, as shown in Figure 5. The pretrained model predicts the action relationship and uses the relationship category as the edge label. In each relational tuple <subject-predicate-object>, the subject and object are the 2048-dimensional attribute feature from the object detection network's RoI pooling. The image region feature corresponding initializes the feature of the predicate to the minimum circumscribing moment of two bounding boxes belonging to the subject and object. The above features are concatenated together and then input to the subsequent classification layer for obtaining the relationship category of the predicate. The *N* × (*N* − 1) relational tuples are input into (excluding self-relations) the relational classification network. Edges with a probability larger than 0.5 are kept to form an action graph, as shown in Figure 6(b).

Yang et al. [73] constructed scene graphs based on reference sentences in the training phase to reconstruct the sentence to accomplish the auto-encode training. The scene graph divides its nodes into three categories: object nodes, relational nodes, and attribute nodes. For each <subject-predicate-object> tuple, the subject and object correspond to the object node *o*_*i*_ and *o*_*j*_. The *l* attribute of the object corresponds to the attribute node *a*_*i*,*l*_, and the relationship between the two objects *i*, *j* corresponds to the relationship node *r*_*ij*_. Each node in the scene graph is represented by a feature vector of *e*_*o*_, *e*_*a*_, *e*_*r*_ ∈ *R*^*d*^, respectively. The object node *o*_*i*_ and all of its attribute nodes *a*_*i*,*l*_ have connections by an edge from the object node to the attribute node. If there is a relationship node, the subject-object node *o*_*i*_ will first connect to the relationship node *r*_*ij*_, and then the relationship node *r*_*ij*_ will connect to the object object node *o*_*j*_. The constructed graph is shown in Figure 6(c). In terms of implementation, they adopt the scene graph constructor used in [83] first to convert sentences into syntactically independent trees and then convert the trees into scene graphs according to the rules mentioned in [75].

Chen et al. [74] designed a customized captioning model to generate sentences according to an abstract graph. The abstract graph is a scene graph customized according to the user's wish. The different forms of description graphs determine the level of detail in the generated caption. Specifically, the abstract graph is constructed by the combination of three types of nodes: (1) object nodes, (2) attribute nodes (representing a specific attribute of an object node), and (3) relationship nodes. The construction of the abstract graph is to add the nodes and edges into the graph according to the user's interests. Specifically, given all *N* object boxes of an image, if the user wants to know the content of the *i* object box, the object node *o*_*i*_ is added to the abstract graph. At the same time, if the user wants to know about the attribute characteristics contained in the object node *o*_*i*_, *l* attribute nodes are added, and each attribute node corresponds to a path from *o*_*i*_ to *a*_*i*,*l*_ directed edges. If the user wants to describe the relationship between two objects, add the corresponding relationship node *r*_*i*,*j*_ in the abstract graph, and build the edge connection between the subject and the object. The subject-object node *o*_*i*_ points to the relationship node *r*_*i*,*j*_, and then the relationship node *r*_*i*,*j*_ points to the object object node *o*_*j*_. The features corresponding to the object nodes and attribute nodes in the abstract graph adopt the visual features of the corresponding object bounding box. The extraction method for the relational node is mainly used to extract the union frame features of two objects. The result of its construction is shown in Figure 6(d).

In summary, the graph method represents more complex action relationships between objects than the tensor method. At the same time, some unnecessary relationship information is also eliminated, which can better retain important relationship content. There has also been a more significant improvement in computational cost and model performance. But the disadvantage is that it depends on the effectiveness of the relationship detection network and relies on training additional relationship information, which increases the complexity of the entire process. In the geometric graph, each edge represents a certain orientation. But in the semantic graph, each edge directly corresponds to a relational category. This more detailed representation of the relationship makes the semantic graph more effective to model the alignment of relational words. However, the limited number of relational categories also limits the variety of generated relational words. At the same time, the semantic similarity between different categories is also eliminated due to the classification operation.

### 3.3. Relational Encoding

For a different type of relational data structure, the encoding methods can be divided into two methods: (1) tensor-based method and (2) graph-based method. This section mainly focuses on different relational encoding methods used in relational captioning.

#### 3.3.1. Tensor-Based Method

The tensor-based method is adopted when the positional relation information or the action relation information is extracted as a relation feature tensor. In this case, each image will correspond to a relational feature tensor *N* × *N* × *d*. If it is a geometric feature tensor between objects, then *d* is of size 4. And if it is the relational feature tensor extracted from the relational action information between objects, then the data of *d* depend on the dimension of the model.

#### 3.3.2. Geometric Multiplier

**Figure 7:**
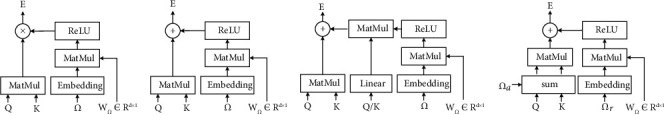
Geometric tensor methods.

For the geometric tensor, Herdade et al. [62] used the tensor as a multiplier to adjust the attention weight in the self-attention of the encoder side. In Section 2, the weight calculation in the self-attention operator relies on the similarity between the query vector and the critical vector. The geometric tensor, the prior information of the positional relationship between objects, is used to adjust each weight element in the self-attention operator. Herdade et al. [62] use the following formula:

(12) $$\begin{array}{c} \omega_{G}^{i,j}=ReLU\left(Emb\left(\lambda \left(i,j\right)\right)W_{G}\right)\text{,} \end{array}$$

where *λ*(*i*, *j*) represents the (*i*, *j*)th vector in the geometric tensor. Emb is an embedding layer, which first maps the geometric vector of 4 dimension to high-dimensional feature space and then calculates each element's positional information through sinusoidal position encoding. Finally, the *d*-dimensional vector is transposed to a scalar factor through *W*_*G*_, and negative values are filtered through the ReLU activation function. Noted that the attention weight *E* in the self-attention operator describes the similarity of *i*-th and *j*-th objects in each element, which is the same as the geometric tensor (describing the positional information of *i*-th and *j*-th objects). As a result, taking *ω*_*G*_^*i*,*j*^ as the scaling factor, adjust the element with the same *i* and *j* indexes in the attention weight *E*. The formula is shown as follows and Figure 7(a) shows the framework:

(13) $$\begin{array}{c} \omega^{i,j}=\frac{\omega_{G}^{ij}\exp\left(\omega_{A}^{ij}\right)}{\sum_{l=1}^{N}\omega_{G}^{il}\exp\left(\omega_{A}^{il}\right)}\text{.} \end{array}$$

The geometric multiplier is designed to modulate the attention weight between each pair of objects for introducing the prior positional knowledge. Each value of the conventional attention weight *E* is like the similarity between *i*-th and *j*-th objects. With the shape identity, each value of the geometric tensor is assigned to the corresponding value with the same index in the attention weight. It is an ingenious and convenient way to introduce positional information in interactive learning. However, the effectiveness of generating better sentences is agnostic and uncontrollable.

#### 3.3.3. Geometric Bias

In addition to scaling the similarity between the *i*-th and the *j*-th object in the weight matrix, Guo et al. [39] adopted a biased method to adjust attention weight. Specifically, the geometric tensor passes through a series of functions and is added to the original weight matrix as a deviation. Guo et al. [39] designed 3 functions for three types of geometric bias: (1) content-independent geometric bias, (2) query-dependent geometric bias, and (3) key-dependent geometric bias. The content-independent geometric bias is reasoned from the geometric tensor and is independent of the visual content. The geometric tensor is transformed into a scalar through a learnable parameter *w*_*g*_^*T*^. Then, it is directly added to the weight in the self-attention operator after being filtered by the ReLU nonlinear function. As shown in Figure 7(b), its calculation formula is as follows:

(14) $$\begin{array}{c} G_{ij}=ReLU\left(FC\left(f_{ij}^{g}\right)\right)\text{,}\\ E=QK^{T}+ReLU\left(\omega_{g}^{T}G\right)\text{.} \end{array}$$

Unlike the independent bias, the query-dependent and key-dependent geometric biases take a further step to compute the similarity with the visual query or key. As shown in Figure 7(c), the specific calculation method is as follows:

(15) $$\begin{array}{c} E=QK^{T}+Q^{\prime T}G\text{,}\\ E=QK^{T}+K^{\prime T}G\text{.} \end{array}$$

Compared with the previous method, Luo et al. [83] used the geometric tensor, including the absolute position geometric tensor and the relative position geometric tensor. The absolute position geometric tensor is directly added to the query vector and key vector as the position feature vector, and the relative position geometric tensor is added as the deviation of the attention weight *E*. As shown in Figure 7(d), the calculation formula is as follows:

(16) $$\begin{array}{c} E=\frac{\left(Q+pos_{q}\right){\left(K+pos_{k}\right)}^{T}}{\sqrt{d_{k}}}+\log\left(\Omega \right)\text{,} \end{array}$$

where **pos**_*∗*_ is the absolute position geometry tensor corresponding to each element in the query vector or key vector. Ω is the relative position geometry tensor. Like the multiplier method, the tensor-based process uses each element of the geometric tensor to function on the element of the attention weight with the same position. This method is straightforward and effective but less interpretable.

### 3.4. Graph-Based Methods

The graph-based method is specific to processing the graph data. The graph-structured data filter some unreasonable relationships through the prior knowledge learned in the pretrained model.

#### 3.4.1. Label-Aware GCN

**Figure 8:**
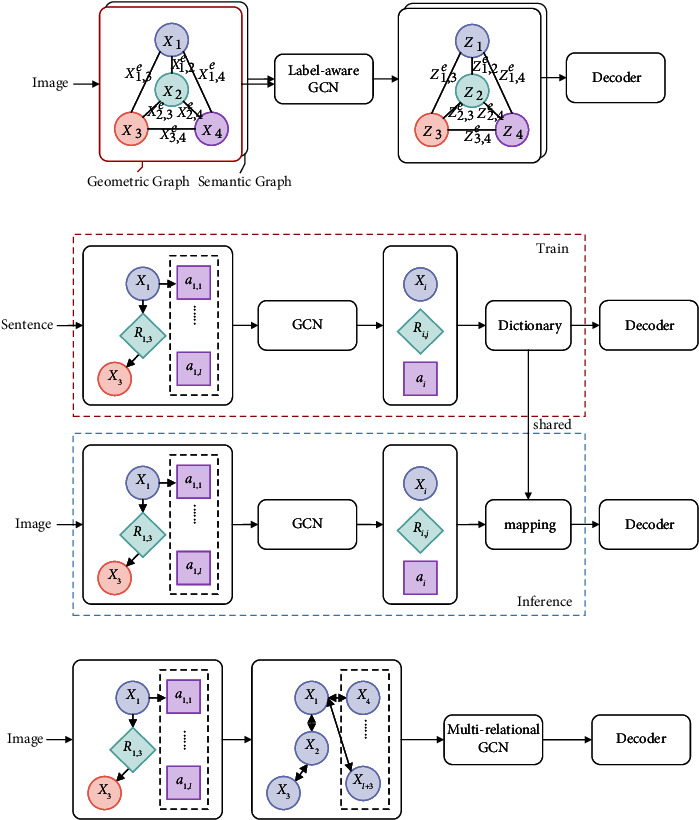
Graph-based methods: (a) label-aware GCN; (b) SGAE; (c) multirelational GCN with customized abstract graph.

Yao et al. [72] designed a graph convolutional network to take the knowledge from the labeled edge and its direction (Figure 8). Each node considers all the connected labeled edges to fuse the relational label and its connected nodes.

Specifically, each image can be transformed into a semantic and positional graph to represent the motion and position relation. The semantic graph is directed, and its edges are labeled with the action relationship. The positional graph is an undirected graph with labeled edges. To make the graph convolutional network aware of the edge's label and its direction, each layer is designed as follows:

(17) $$\begin{array}{c} v_{i}^{t}=\rho \left(\underset{v_{j}\in N\left(v_{i}\right)}{\sum }g_{v_{i},v_{j}}\left(W_{di\;r\left(v_{i},v_{j}\right)}v_{j}+b_{lab\left(v_{i},v_{j}\right)}\right)\right)\text{,}\\ g_{v_{i},v_{j}}=\sigma \left({\tilde{W}}_{dir\left(v_{i},v_{j}\right)}v_{j}+{\tilde{b}}_{lab\left(v_{i},v_{j}\right)}\right)\text{,} \end{array}$$

where *W*_di r(*v*_*i*_, *v*_*j*_)_ selects different transformation matrices according to the type of each edge. Specifically, if the *i* object *v*_*i*_ is the subject in a relation tuple <subject-relation-object>, then the transformation matrix is *W*_1_; if the *i* object *v*_*i*_ is the object, then the transformation matrix becomes *W*_2_. Similarly, when dealing with the self-connected edge, the transformation matrix is set to be *W*_3_. lab(*v*_*i*_, *v*_*j*_) represents the category of the edge. *g*_*v*_*i*_,*v*_*j*__ is a weight function to determine the importance of the edge in the calculation. Compared with the conventional GCN, the label-aware GCN introduces the relationship information in each edge with the corresponding relational label. The label triggers the embedding function to form the edge features to fuse the connected nodes' relational information further. By introducing the graph, the connection between nodes determines the interactive learning and guides the model to generate the content between corresponding objects. It is more explainable than the geometric methods, which use the full-connected graph.

#### 3.4.2. Scene Graph Auto-Encoder

Yang et al. [73] proposed the Scene Graph Auto-Encoder (SGAE) model to learn a recoder to optimize the original visual features through reconstruction of the sentence in training. The scene graph is constructed from the ground-true sentence, and each visual feature further fuses features according to the connection in the graph. It is shown in Figure 6(c), which includes object nodes, relational nodes, and attribute nodes.

(18) $$\begin{array}{c} x_{r_{ij}}=g_{r}\left(e_{o_{i}},e_{r_{ij}},e_{o_{j}}\right)\text{,}\\ x_{a_{i}}=\frac{1}{Na_{i}}\overset{Na_{i}}{\underset{l=1}{\sum }}g_{a}\left(e_{o_{i}},e_{a_{il}}\right)\text{,}\\ x_{o_{i}}=\frac{1}{Nr_{i}}\underset{o_{j}\in <o_{i}-r_{i*}-o_{*}>}{\sum }g_{s}\left(e_{o_{i}},e_{r_{ij}},e_{o_{j}}\right)+\underset{o_{k}\in <o_{*}-r_{*i}-o_{i}>}{\sum }g_{o}\left(e_{o_{k}},e_{r_{ki}},e_{o_{i}}\right)\text{,} \end{array}$$

where *x*_*r*_*ij*__ is the node feature of the relation node *r*_*ij*_, and its neighbor node features *e*_*o*_*i*__, *e*_*r*_*ij*__, and *e*_*o*_*j*__ belong to the corresponding node in the relation tuple <*o*_*i*_-*r*_*ij*_-*o*_*j*_>. *x*_*a*_*i*__ represents the attribute information of the *i* object node, and its neighbor *e*_*o*_*i*__ and *e*_*a*_*il*__ belong to the object node *i* and *l*-th attribute feature. An object may have multiple attributes, each attribute corresponds to an attribute node. *N* is the total number of all attributes. *x*_*o*_*i*__ represents the feature of the *i*-th object node, <*o*_*i*_-*r*_*i∗*_-*o*_*∗*_> represents all the tuples whose *i*-th object as the subject. <*o*_*∗*_-*r*_*∗i*_-*o*_*i*_> represents all the tuples whose *i*-th node is the object. After passing the abovementioned embedding, they use the form of a memory network to set up a dictionary matrix **D** ∈ *R*^*d*×*V*^ to optimize the input node feature *x*. The calculation formula is as follows:

(19) $$\begin{array}{c} \hat{x}=Dsoftmax\left(D^{T}x\right)\text{.} \end{array}$$

The optimized feature $\hat{x}$ is input to the subsequent decoder to regenerate the sentence and compare with the real input sentence. The error is fed back to the network for self-encoding training. The auto-encoder method uses the reconstruction to learn the semantic knowledge which begins from the sentence and regenerates it. The semantic knowledge reflects in the scene graph and assists the inference process. The whole framework is shown in Figure 8

#### 3.4.3. Multirelational GCN

Chen et al. [74] proposed a customized abstract graph to generate specific captions. For representing each node, the features of the object nodes and attribute nodes adopt the visual features of the corresponding object bounding box, which are reasoned from the object detection network. The union bounding box's feature of two objects is used for the relational node. At the same time, Chen et al. made various types of nodes corresponding to different transformation matrices in feature embedding to further distinguish different kinds of nodes. The formula is shown as follows:

(20) $$\begin{array}{c} x_{i}^{\left(0\right)}=\left\{\begin{array}{cc} v_{i}⊙W_{r}\left[0\right]\text{,} & \text{if }i\in 0\text{;}\\ v_{i}⊙\left(W_{r}\left[1\right]+pos\left[i\right]\right)\text{,} & \text{if }i\in a\text{;}\\ v_{i}⊙W_{r}\left[2\right]\text{,} & \text{if }i\in r\text{;} \end{array}\right. \end{array}$$

where *W*_*r*_[*k*] is the transformation matrix and its three matrices corresponding to three types of nodes. pos[*i*] adds the order information for different attribute nodes *a*_*i*,*l*_. According to the abovementioned embedding methods, the features of each node in the abstract graph are fused with their adjacency nodes. Meanwhile, the directed abstract graph is converted into an undirected graph which fits with the GCN. Chen et al. [74] designed a multirelational GCN (Figure 8) so that graph convolution learns different sets of parameters according to the edge types. There are six different types of edges: (1) object node to attribute node, (2) subject node to relational node, and (3) object node to relational node point and their inverse edges. The transformation transforms the direct graph into a unidirected graph and feeds into the multi-relational GCN to refine each node's feature. Different transformation matrices in each layer of the graph convolutional network are used to map the edges of different categories. Specifically, each layer is calculated as follows:

(21) $$\begin{array}{c} x_{i}^{l+1}=\sigma \left(W_{o}^{l}x_{i}^{l}+\underset{r\in R}{\sum }\underset{j\in N}{\sum }\frac{1}{N}W_{r}^{l}x_{j}^{l}\right)\text{,} \end{array}$$

where *l* represents the different layers in the graph convolutional network, the parameters for different classes of edges in each layer are shared. Through stacking encoders, each node feature is learned according to the connection between the nodes in the graph. The multirelational GCN is based on the abstract graph, which the user designs for generating the customized caption. The controllable ability has been improved, and the abstract graph determines the attribute, object, and relationship feature fed into the model.

In summary, Table 1 summarizes the methods used in relational feature construction and relational encoding by current methods in relational captioning.

## 4. Dataset and Evaluation

### 4.1. Dataset

The main datasets used in relational captioning are the following 4 datasets: (1) VisualGenome [84]; (2) MSCOCO [85]; (3) Flickr8K [86]/Flickr30k [87]; (4) PASCAL 1K [7].

#### 4.1.1. VisualGenome

There are 108K images in total and many object annotations, attribute information annotations, and relationship annotations between objects for tasks such as object detection and visual relationship detection. In relational captioning, it is mainly used as a pretraining dataset to pretrain the object detection or the visual relationship detection network. In the pretraining stage, the training, validation, and test dataset split is followed by Anderson et al. [5]. Specifically, 98K images are used for training, and the remaining 10K images are divided into validation and test sets, respectively. When Yao et al. [72] pretrained the target detection network, the dataset was filtered to retain 1600 object categories and 400 attribute categories. When dealing with pretrained object detection networks, it mainly selects the top 50 standard action relationships and artificially classifies them into 20 categories.

#### 4.1.2. MSCOCO

The Microsoft COCO Captions dataset [85] is developed by Microsoft Team with the goal of scene understanding, capturing images from complex scenes, and can perform multiple tasks such as image recognition, segmentation, and captioning. The dataset uses Amazon's “Mechanical Turk” service to manually generate at least five sentences for each image. It contains more than 1.5 million sentences. The training set contains 82,783 images, the validation set contains 40,504 images, and the test set contains 40,775 images. In captioning tasks, the “Karpathy” split [5] is the standard data split method, which takes 5000 images in the validation set for evaluation and 5000 images for testing. The rest of the training and validation datasets are used for training.

#### 4.1.3. Flickr8K/Flickr30k

Flickr8k [86] images are from Yahoo's photo album website Flickr, including 8,000 images, 6,000 images for training, 1,000 for evaluation, and 1,000 for testing. Flickr30k [87] contains 31,783 images collected from the Flickr website, mainly depicting human engagement. The manual label corresponding to each image is still five sentences.

#### 4.1.4. PASCAL 1K

It is a subset of the well-known PASCAL VOC challenge image dataset [7], which provides a standard image annotation dataset and a standard evaluation system. The PASCAL VOC dataset consists of 20 categories. Amazon's Turk Robot service was then used to label each image with five descriptions manually. The dataset has the excellent image quality and complete annotation, which is suitable for testing algorithm performance.

### 4.2. Evaluation

The evaluation standard of relational captioning is consistent with the standard evaluation used in natural language processing to evaluate the similarity between the generated sentence and the ground-truth sentence. The evaluation metrics: BLEU [88], METEOR [89], ROUGE [90], CIDEr [91], and SPICE [92]. For the five metrics, BLEU and METEOR are used for machine translation, ROUGE for automatic translation summaries, and CIDEr and SPICE for image captioning. In principle, the abovementioned evaluation metrics measure the n-gram consistency between generated sentences and reference sentences and are also affected by the importance and rarity of n-grams in the corpus.

#### 4.2.1. BLEU

As a widely used and essential evaluation metric in machine translation, BLEU [88] mainly measures the degree of the repetition between the generated sentence and the reference sentence. The number of identical n-grams in both generated and reference sentences determines the BLEU score. With the more significant number, the BLEU score is higher, meaning the generated sentences are closer to the reference sentences. With the increase of the *n* in n-gram, BLEU considers the correlation no longer limited to several words but prefers the correlation between contents. The higher the BLEU score, the better the generated sentences.

#### 4.2.2. METEOR

METEOR [89] mainly considers the influence of synonyms and word forms in comparing generated sentences with all reference sentences. When evaluating the fluency of the sentence, METEOR is computed based on the chunks, which are constructed by considering the combination of semantically consecutive words. The word's consistency between the candidate and reference sentences is measured by the chunk. At the same time, METEOR is calculated by combining the precision, recall, and F-values of matching various cases. The higher the METEOR score, the better the sentence performance.

#### 4.2.3. ROUGE

ROUGE [90] is a set of evaluation metrics designed to evaluate text summarization. ROUGE-L is used in relational captioning. It is calculated using the longest common subsequence between the generated and reference sentences. The score is calculated by summing the recall and precision of the longest common subsequence. The higher the ROUGE score, the better the sentence performance.

#### 4.2.4. CIDEr

CIDEr [91] is an evaluation metric specially designed for captioning. It measures the consistency of image annotations by performing a term frequency-inverse document frequency (TF-IDF) weight calculation for each n-gram. This metric treats each sentence as a “document,” represented as a TF-IDF vector, and then computes the cosine similarity between the generated sentence and the reference sentence. This indicator makes up for a shortcoming of BLEU, in which all words on the match are treated equally. Meanwhile, it considers the importance of the information of each word itself. Likewise, the higher the CIDEr score, the better the performance.

#### 4.2.5. SPICE

SPICE [92] is a semantic evaluation metric for image captions, which measures how effectively image captions recover objects, attributes, and relationships between them. On the image captioning dataset, SPICE can better capture human judgments of model captions than existing n-gram metrics.

Table 2 shows the scoring index ranking of the models used in the current relational image description on the MSCOCO dataset.

## 5. Conclusion

This paper mainly summarizes the procedure of relational captioning and the development of each part in recent years. The relational captioning further focuses on the relationship between objects in the image. By introducing and incorporating the relationship information, the sentences generated by the model have better sufficiency and accuracy. We summarize the framework used in relational captioning and divide the relational procedure into two parts: feature construction and feature encoding. Combined with the characteristics of the relationship between objects, the relationship is further divided into the positional relationship and action relationship. The methods used for learning each relationship are discussed in the feature construction and encoding stages. In addition, we also summarize the datasets commonly used in relational captioning and the related evaluation metrics of the model.

We conclude by summarizing the current challenges in relational caption and clarifying our vision for this aspect. There are two main challenges in relational captioning, which is existed in feature construction and feature encoding. In terms of feature construction, it is challenging to find an appropriate method which considers as many relationship categories as possible while satisfying the content correlation between each relationship category on the textual modality. Second, in terms of feature encoding, it is challenging to make the feature perceive the semantic difference of various relational information and maintain its original visual knowledge. According to the abovementioned two challenges, we believe that future work has the following space for improvement in relational captioning:

1. The feature construction of positional relationships is mainly limited to the handmade geometric feature extracted from objects' bounding box in 2-dimensional space. The geometric feature is susceptible to the scale of the object box.
2. The feature of motional relationship depends on the performance of the pretrained feature extracted network. Better features can be obtained by adjusting the training objectives of the pretrained network in upstream tasks.
3. About feature encoding, the current cross entropy or reinforcement learning training objectives make it difficult for the features output by the encoder to fully reflect the differences between different relationship categories while retaining visual knowledge. Compared with the end-to-end training method, the current pretraining-finetuning method [67–69] could use specialized objective function to obtain more powerful features.
4. The alignment between relational features and relational vocabulary is ambiguous. The generation of relational vocabulary mainly depends on the global image information instead of relational features.

## Figures and Tables

**Table 1:** Summary of the various methods in the relational captioning.

Methods	Feature construction	Relational encoding	Decoder
GCN-LSTM [72]	Positional relation: directed graph with label	Convolutional graph network	Two-LSTMs decoder
	Motional relation: directed scene graph		
SGAE [73]	Motional relation: directed scene graph	Auto-encoder	Two-LSTMs decoder
ORT [62]	Positional relation: directed graph with label	Attention multiplier	Transformer
NG-SAN [39]	Positional relation: directed graph with label	Attention bias	Transformer
DLCT [83]	Positional relation: directed graph with label	Attention bias	Transformer

**Table 2:** The evaluation scores of relative caption methods on COCO “*Karpathy*” test split.

Methods	B-1	B-4	M	R	C	S
GCN-LSTM [72]	80.5	38.2	28.5	58.3	127.6	22.0
SGAE [73]	80.8	38.4	28.4	58.6	127.8	22.1
ORT [62]	80.5	38.6	28.7	58.4	128.3	22.6
NG-SAN [39]	—	39.9	29.3	59.2	132.1	23.3
DLCT [83]	81.4	39.8	29.5	59.1	133.8	23.0

## Data Availability

This paper is an overview paper in which the data reported are derived from corresponding published research studies. These prior studies (and datasets) are cited at relevant places within the text as references.
